# HIV sero-discordance among married HIV patients initiating anti-retroviral therapy in northern Vietnam

**DOI:** 10.1186/s12981-016-0124-9

**Published:** 2016-11-15

**Authors:** Vu Van Tam, Do Duy Cuong, Tobias Alfven, Ho Dang Phuc, Nguyen Thi Kim Chuc, Nguyen Phuong Hoa, Vinod Diwan, Mattias Larsson

**Affiliations:** 1 Division of Global Health, Department of Public Health Sciences, Karolinska Institute, Stockholm, Sweden; 2 Health System Research Project, Ha Noi Medical University, Ha Noi, Vietnam; 3 Oxford University Clinical Research Unit, Ha Noi, Vietnam; 4 Department of Probability and Mathematical Statistics, Institute of Mathematics, Ha Noi, Vietnam

**Keywords:** HIV, ART, Gender, Late presentation, Sero-discordance, Vietnam

## Abstract

**Background:**

In many countries in Asia, the HIV epidemic is in a concentrated phase, with high prevalence in certain risk groups, such as men who inject drugs. There is also a rapid increase of HIV among women. The latter might be due to high levels of sero-discordant couples and increasing transmission from male to female partners over time.

**Methods:**

All adult married patients initiating antiretroviral treatment at four out-patient clinics in Quang Ninh province in north-eastern Vietnam between 2007 and 2009 were asked to participate in the study. Clinical information was extracted from patients’ records, and a structured questionnaire was used to collect social, demographic and economic data.

**Results:**

Two hundred eighty-eight married patients for whom information on the HIV status of their spouse was available were included in the study. Overall, the sero-discordance rate was 58%. The sero-discordance rate was significantly higher among married males, 71% had spouses not infected, than married females, of whom 18% had spouses not infected. Other factors associated with a high rate of sero-discordance were injection drug use (IDU) history, tuberculosis (TB) history and the availability of voluntary counselling and testing (VCT) in residential locations. High sero-concordance was associated with college/university education.

**Conclusion:**

The sero-discordance was significantly higher among married males than married females. Other factors also related to high sero-discordance were history of IDU, history of TB and the availability of VCT in residential locations. In contrast, college/university education and female sex were significantly related to low sero-discordance. To contain the increasing HIV prevalence among women, measures should be taken to prevent transmission among sero-discordant couples.

*Trial registration* NCT01433601

## Background

In couples where one person is living with HIV and the other is HIV negative, the HIV-negative partner is at continuous risk for infection. Sexual behaviour, viral load and sexually transmitted disease influence the risk for transmission between partners. In most Asian countries, including Vietnam, the HIV epidemic is in a concentrated stage, with the highest HIV prevalence among certain risk groups, such as people who inject drugs (PWID), of whom the majority are men [1]. In areas with a generalised HIV epidemic, as in many sub-Saharan African countries, the HIV prevalence is higher among women than men [2, 3]. However, increasing numbers of women are contracting HIV also in settings with concentrated HIV epidemics [2]. In Vietnam, the first HIV case was detected in 1990. By March 2010, the HIV prevalence among adults aged 15–49 years was estimated to be 0.53%, with 209,424 HIV+ cases reported, of whom 45,227 had died [4]. Since late 2005, antiretroviral therapy (ART) programs have been scaled up, with funding coming mostly from the U.S. President’s Emergency Plan for AIDS Relief and the Global Fund to Fight AIDS, Tuberculosis and Malaria.

The aim of this project was to study sero-concordance and discordance rates in a cohort of people living with HIV initiating ART who were recruited from 2007 to 2009. A further aim was to study factors influencing discordance and concordance, including gender.

## Methods

### Study setting

The study was carried out at 4 out-patient clinics (OPCs) in Quang Ninh, a province in north-eastern Vietnam with a population of 1.1 million. Quang Ninh’s economy is growing rapidly, based mainly on industries such as coal mining, cement manufacturing and harbours, as well as tourism associated with the famous Ha Long Bay. Quang Ninh is also among the areas hardest hit by the HIV epidemic in Vietnam, with an estimated HIV prevalence of slightly above 1% among those aged 15–49 years [5].

### Study participants

Of 660 naïve HIV positive patients eligible for ART who were registered continuously from July 2007 to November 2009 at 4 OPCs in Quang Ninh, Vietnam, 640 patients (97%) gave their consent to participate in a Randomized Controlled Trail (RCT) to assess the effect of peer support on virological treatment failure rates. Of the patients 288 (46%) were married. For the purpose of this study, we included only married patients whose partners were willing to provide their HIV test results. Among these 452 were male (70.6%) and 188 female (29.4%).

Patients in the intervention group received enhanced adherence support from peer supporters and the control group had conventional support. According to the Vietnamese National HIV Guidelines (2005), HIV-infected individuals were eligible to be registered for free at an ART clinic if they: (1) were confirmed HIV positive, (2) possessed valid civil registration information (home address and telephone), (3) had a family member who can act as a supporter, and (4) agreed to enroll and to be followed up in the ART program. Each clinic was staffed with two medical doctors, one adherence counselor, one reception nurse, one phlebotomy nurse, one pharmacist, and one volunteer who was a person living with HIV (PLHIV). The staff were trained on basic and advanced HIV care and treatment and certified by Ministry of Health.

### Data collection

Before initiating ART, each patient was interviewed by a trained interviewer using a structured questionnaire. The questionnaire was designed to collect information about family structure, socioeconomic situation, mode of HIV transmission, history of HIV diagnosis, TB and ART, as well as alcohol and heroin use. The partners of married patients were encouraged to take a HIV test provided free under the OPC’s VCT program. Clinical staging and baseline laboratory tests (including a full blood count, alanine aminotransferase, hepatitis coinfection, CD4+ T cell count and plasma HIV viral load) were collected from the participants’ medical records. The viral load was determined using a method based on the enzyme-linked immunosorbent assay (Exavir™ Load) [6]).

### Statistical analysis

The characteristics of the enrolled patients were described and grouped first in relation to the HIV status of their wife/husband. Univariate and multivariate analyses were carried out to study the effect of occupation, education level, resident’s area and gender on the HIV sero-status of the patient’s spouse or partner. Other indicators assessed included the following: CD4 count, clinical staging history of TB infection and injecting heroin and known duration of HIV infection between HIV diagnosis and initiating ART.

The collected data were analysed using SPSS version 20. Chi Square tests were performed to examine the difference between proportions, with 95% confidence intervals (CI) used in the comparisons. Odds ratios (ORs) were calculated with 95% CI to compare risks between the two patient groups. Multiple logistic regression equations were used to compute the ORs of each factor adjusted for other factors presented in given models (aORs). In all the tests and the regression models, *p* values less than 5% were considered significant.

### Ethical considerations

The study was approved by the Institutional Review Boards of Hanoi Medical University, Ministry of Health, Vietnam (numbers 26/IRB, 66/HMURB, 59/HMURB and 98/HMURB) of Hanoi Medical University) and the Regional Board for Ethics Review from Karolinska Institutet in Stockholm, Sweden (number 2006/1367-31/4). Before conducting the study, written informed consent was obtained and all identifying information (names, initials, etc.) were then omitted to ensure the confidentiality. Patients in the intervention group were informed about the study and that they would be visited by a peer supporter and that the home visits might disclose their HIV status. In order to avoid unintentional disclose of HIV status through study participation efforts were made. The peer supporters where instructed to act in a manner that would not evoke suspicion in the study participants neighborhood. In advance of each visit the peer supporters called the study participants to decide when and where to meet. The study participants could at any time cancel a meeting or decide not to participate in the intervention or study. Biological samples were collected and used only after written consent. All blood samples were coded to protect the identity of patients and to ensure confidentiality.

Both intervention and control groups received equal care and treatment and those who did not fulfill the inclusion criteria’s for the study were managed with a set standard of care and treatment according to the National HIV Treatment Guidelines. Peer-supporters received ART at the study clinics and were treated in the same way as other HIV patients according to the National Guidelines. Moreover, peer-supporters’ viral load (ExaVir^TM^ Load) were also measured every 6 months, and they received a salary and transportation fees each month. Data were accessible only to research team, data manager and coordinator of the project.

## Results

### Social, demographic and economic characteristics

Among the 288 married patients who were ART-naive and where information on the HIV status of their spouses was available, the majority were HIV-infected men (75%), and the mean age was 32 (SD = 5.5) years (Table 1). The mode of HIV transmission reported among men was mainly sharing needles during heroin use (58%), whereas sexual transmission was reported in 94% of women (*p* < 0.01). IDU, past or present, was reported by half of the patients, significantly higher among males (63%) than females (4.2%) (*p* < 0.01). Of these patients, 18% reported active heroin use in the last 6 months.

**Table 1:** Patients’ socio-economic and clinical-laboratory characteristics at enrolment

Characteristics	Sub-character	Females (*n* = 72)		Males (*n* = 216)		*p* value*
		(%)	95% CI	(%)	95% CI	
Education	Secondary or less	51	39–63	48	41–55	>0.05
	High school	36	25–48	42	35–49	
	College/university	13	6–22	11	7–16	
Occupation	Unemployed	12	8–17	27	23–32	<0.05
	Employed	88	80–95	73	68–77	
Known PLHIV in family	Yes	65	59–72	27	23–31	<0.01
	No	35	28–42	73	69–78	
Mode of HIV infection	Sexual intercourse	94	89–98	29	24–33	<0.001
	IDU	4	2–8	64	59–68	<0.001
	Do not know	2	0–5	7	5–10	
Disclosure of HIV status	Yes	92	88–95	92	91–96	>0.05
	No	8	4–11	8	5–10	
History of IDU	Yes	5	2–8	73	69–78	<0.001
	No	95	92–96	27	23–31	
Clinical stage	Stage 3 or 4	33	28–39	61	58–64	<0.05
	Stage 1 or 2	67	62–70	39	35–44	
Duration of known HIV infection	≥2 years	36	30–41	45	40–49	>0.05
	<2 year	64	57–70	55	50–60	
CD4 cell count	≥100/µl	65	58–71	45	39–51	<0.01
	<100/µl	35	29–41 159 159	55	50–60 99 60	
CD4 cell count by group (cells/µl)	0–49 50–99 100–149 150–199 ≥200 Mean Median	18.1 12.5 16.7 22.2 30.6 159 159		46.8 18.1 9.7 11.6 13.9		<0.0001
Viral load by group	<400 400–3499 3500–9999 10000–49999 ≥50000	0 16.2 6.5 25 52.3		0 20.8 12.5 26.4 40.3		>0.05

* Chi square test comparison between proportions of males and females

A large majority of the patients (92%) had disclosed their HIV+ status to other people (spouse, parents, siblings).

### Clinical characteristics

Clinical stage 3 or 4 (AIDS defining Opportunistic Infections) at diagnosis was significantly (*p* < 0.01) more common among males than females (61 vs. 34%). Males were significantly (*p* < 0.001) more immunosuppressed than females, and the average CD4 counts was (104/µl) and (152/µl), respectively.

### Sero-discordance

Overall, the sero-discordance rate was 58%. Men had a significantly (*p* < 0.01) higher rate of sero-discordance compared to women, 71 and 18%, respectively. Males reporting IDU had significantly higher sero-discordance (*p* < 0.001) compared to males not reporting IDU (79 vs. 58%). Among women reporting IDU compared to women not reporting IDU the observed effect size was lager then among men (33 vs. 17%), however, however no significant difference in sero-discordance.

## Discussion

There was a high sero-discordance rate among the married couples in our study, and it was higher among men than among women. The sero-discordance rate shown in this study is higher than that reported in other countries, e.g. Thailand (58%) [7] or Nigeria (25%) [8].

High sero-discordance rate was associated with male sex, history of IDU and diagnosis of TB. Patients with a history of IDU might be less sexually active, hence posing less of a risk of transmitting the virus to their spouse, also shown in an earlier study [9]. Patients with a history of IDU were commonly diagnosed early as part of the on-going sentinel surveillance program among risks groups. This may explain the counterintuitive result that patients with known HIV infection for more than 2 years had a higher sero-discordance rate than patients with known infection less than 2 years. Early HIV detection through sentinel surveillance or VCT before development of severe immunosuppression or indication for ART might sensitise the patients to preventive measures such as condom use or abstinence, as shown in a study from Ethiopia [10]. In Dong Trieu, where no sentinel surveillance activities or VCT were available until 2002 compared to 1995 in Ha Long City, the sero-concordance rate was significantly higher.

As shown, patients with higher education had a significantly lower sero-discordance rate. This could be due to a more favourable social status in the community, making the individual more socially vulnerable to accidental disclosure and stigmatisation. This factor may be a strong disincentive to HIV testing [11, 12], thereby delaying diagnosis and increasing the duration of exposure and risk for HIV transmission to the partner. In Vietnam, a high proportion of men have visited female sex workers [13]. As the latter are a vulnerable group with a high prevalence of HIV and Sexually Transmitted Diseases (STDs) [14], the male partners may have been infected not only with HIV, but also STDs such as syphilis, herpes type 2 and gonorrhoea, all of which increase the risk for HIV transmission [15].

There was a much higher rate of sero-discordance among male married patients than female married patients. A study in Italy concluded that the efficiency of male-to-female transmission was 2.3 times greater than that of female-to-male transmission [16]. Thus, a higher discordance rate would be expected among married male patients with an equal sex distribution of index patients. This factor, combined with the finding that men were more severely immunosuppressed than women, indicating a longer duration of HIV infection, may lead to the conclusion that the male partner was the index case in most cases and infected their female partner. This idea is also supported by the high proportion of widows (40%) in the study population.

High plasma viral load that may increase the risk of transmitting HIV to their sexual partners [17]. This idea is supported by the general trend of increasing HIV prevalence among women, particularly those who live in areas with a high prevalence of male PWID [18, 19]. In our cohort we could not find a significant correlation between high baseline viral load and level of sero-discordance (Table 2).

**Table 2:** Factors related to HIV sero-discordant/concordant status

Characteristics	Variable	Concordance N = 121		Discordance N = 167		Adjusted odds ratio	
		*N* (%)	95% CI	*N* (%)	95% CI	(95% CI)	*p* value
Sex	Male	62 (29%)	23–35	154 (71%)	65–77	1	
	Female	59 (82%)	71–90	13 (18%)	10–29	4.8 (2.0–11.4)	<0.001
Education	Secondary or less	56 (40%)	32–49	84 (60%)	51–68	1	
	High school	45 (39%)	30–48	71 (61%)	52–70	1.2 (0.6–2.3)	NS
	College/university	20 (63%)	44–78	12 (37%)	22–56	3.2 (1.2–8.7)	0.025
Occupation	Unemployed	14 (27%)	16–41	38 (73%)	59–84	0.8 (0.3–1.9)	NS
	Employed	107 (45%)	39–52	129 (55%)	48–61	1	
Having children	Yes	89 (40%)	34–47	132 (60%)	53–66	1	
	No	32 (48%)	36–60	35 (52%)	40–64	1.3 (0.6–2.7)	NS
Resident’s area	Dong Trieu	25 (68%)	50–81	12 (32%)	19–49	5.2 (1.7–15.4)	0.003
	Uong Bi	20 (47%)	31–62	23 (53%)	38–69	2.5 (0.997–6.5)	0.051
	Yen Hung	16 (46%)	29–63	19 (54%)	37–71	2.4 (0.9–6.8)	NS
	Ha Long	60 (35%)	28–42	113 (65%)	58–72	1	
Mode of HIV infection	Sexual intercourse	85 (60%)	51–68	57 (40%)	32–48	1	
	IDU	26 (21%)	14–29	100 (79%)	71–86	0.4 (0.2–1.1)	NS
	Do not know	10 (42%)	23–63	14 (58%)	37–77	0.6 (0.2–2.0)	NS
Hepatitis coinfection	Yes	29 (32%)	23–42	62 (68%)	54–77	0.6 (0.3–1.3)	NS
	No	92 (47%)	40–54	105 (53%)	46–60	1	
History of IDU	Yes	31 (22%)	16–30	109 (78%)	70–84	0.6 (0.3–1.3)	NS
	No	90 (61%)	52–69	58 (39%)	31–48	1	
Clinical stage	Stage 3 or 4	68 (49%)	40–57	71 (51%)	43–59	1	
	Stage 1 or 2	53 (36%)	28–44	96 (64%)	56–72	0.9 (0.4–1.7)	NS
Duration of known HIV infection	≥2 years	80 (46%)	39–54	93 (54%)	46–61	1	NS
	<2 year	41 (36%)	27–45	74 (64%)	55–73	0.6 (0.3–1.3)	NS
History of TB	Yes	10 (25%)	13–41	30 (75%)	58–87	1	
	No	111 (45%)	39–51	137 (55%)	49–61	0.2 (0.05–0.8)	0.026
CD4 cell count	≥100/µl	73 (52%)	43–60	68 (48%)	40–57	1	
	<100/µl	48 (33%)	25–41	99 (67%)	59–75	1.1 (0.6–2.2)	NS
Viral load	≥100,000 copies/ml	40 (39%)	29–49	63 (61%)	51–70	1.1 (0.5–2.1)	NS
	<100,000 copies/ml	81 (44%)	37–51	104 (56%)	49–63	1	

As all the patients were included in the study at the start of ART, the effect of viral suppression on transmission rates is not a likely cause of the high sero-discordance rate, although this has been shown to be the case in other studies [20]. TB may occur at a modest level of immunosuppression, and individuals diagnosed with TB are tested for HIV. These factors might have led to earlier diagnosis and hence a reduction in the exposure time for the spouses. The deterioration in health, as well as common stigmatisation of TB, may have decreased the sexual activity and hence the exposure of the spouse [9].

The high sero-discordance rates of the HIV+ married men may indicate that a large part of the population was aware of preventive measures, especially in the areas where VCT has been carried out for a longer duration. However, the high number of female spouses at risk of exposure emphasises the need for continued and increased measures to prevent transmission such as Information, Education and Communication programs and provision of condoms in the community to prevent HIV transmission among couples [21, 22]. In Zambia, it has been reported that the ideological stance in the U.S. President’s Emergency Plan for AIDS Relief to promote abstinence and faithfulness before condoms (the ‘ABC’ approach) might have deterred the organisations involved in the implementation of the program from giving information and providing condoms to at-risk populations [23]. The ABC approach has not been so influential in Vietnam where condoms have been promoted as a core part of the prevention program. This may have influenced the comparably high rates of sero-discordance reported, in combination with other factors such as the prevalence of IDU and the rate of sexual HIV transmission.

Early initiation of ART reduce sexual transmission, especially among sero-discordant couples, the incidence of Opportunistic Infections (OIs) and death [24–31]. The risk for transmission is proportional to the viral load in combination with other factors that increase the risk for blood to blood contact as STD’s, genital lesions and anal intercourse. Hence viral load monitoring is important to assess both the effect of treatment as well early detection of increased risk for HIV transmission, especially among sero-discordant couples (27). In seen in Table 2 there were no significant difference in viral load between sero-concordant and discordant or male and females. In many low-income countries, patients are generally diagnosed at a late stage of the disease and the ART coverage is in many places still low [28]. As seen in our study, there was limited access to ART, even for those patients who were severely immunosuppressed and with high viral load. The lack of access was mainly due to the program’s regulation, under which the number of patients started on ART each month was limited to avoid overloading health staff.

There are some methodological considerations in this study. Non-naïve was an exclusion criteria, hence patients that had used ART prior to enrolment were not asked to join the RCT. Therefore, this cohort might not be representative of HIV patients in Vietnam. Indicators such as IDU and history of TB were self-reported, and this might have caused an underestimation. Information regarding number of years married, number of sex partners or sexual intercourse behaviour was not collected. Thus, the data might not represent the magnitude of all predictors of sero-discordance among married patients. As the study included only patients who registered at OPCs, information on patients who have not yet registered is lacking. Thus, the study does not fully reflect the entire characteristics of People Living with HIV in Vietnam.

## Conclusions

There was a high sero-discordance rate among the married patients. Being a female, having education at college/university level and living in an area with late initiation of VCT were predictors of higher HIV sero-concordance, whereas being male, having a history of IDU and TB and living in an area with earlier VCT initiation were predictors of the HIV sero-discordance of the partner. To contain the increasing HIV prevalence among women and prevent transmission among sero-discordant couples, measures should be taken to reduce the HIV viral load and exposure. These include providing increased information, as well as condoms and ART to the HIV-positive partner, regardless of their immune status. Pre-Exposure Prophylaxis (PrEP) might in some cases also be an option to reduce transmission. Viral load monitoring is important to assess the effect of treatment, early detection treatment failure or poor adherence, as well as of increased risk for HIV transmission, especially among sero-discordant couples.

## Abbreviations

HIV: human immunodeficiency virus
PWID: people who inject drugs
IDU: injection drug use
TB: tuberculosis
VCT: voluntary counselling and testing
ART: antiretroviral therapy
OPCs: out-patient clinics
CI: confidence intervals
ORs: odds ratios
STDs: sexually transmitted diseases
ABC: abstinence, be faithful, condoms
PLHIV: people living with HIV
